# Protective Effect of Spore Powder of *Antrodia camphorata* ATCC 200183 on CCl_4_-Induced Liver Fibrosis in Mice

**DOI:** 10.3390/nu12092778

**Published:** 2020-09-11

**Authors:** Yilin Ren, Hua-Xiang Li, Lingxi Zhou, Zhen-Ming Lu, Jinsong Shi, Yan Geng, Zheng-Hong Xu

**Affiliations:** 1 School of Pharmaceutical Sciences, Jiangnan University, Wuxi 214122, China; renyilin@jiangnan.edu.cn (Y.R.); shijs@163.com (J.S.); 2 National Engineering Laboratory for Cereal Fermentation Technology, Jiangnan University, Wuxi 214122, China; zhenming_lu@163.com (Z.-M.L.); zhenghxu@jiangnan.edu.cn (Z.-H.X.); 3 Jiangsu Engineering Research Center for Bioactive Products Processing Technology, Jiangnan University, Wuxi 214122, China; 4 College of Food Science and Engineering, Yangzhou University, Yangzhou 225127, China; lihuaxiangyu@126.com; 5 Key Laboratory of Industrial Biotechnology of Ministry of Education, School of Biotechnology, Jiangnan University, Wuxi 214122, China; furenlingxi@163.com

**Keywords:** *Antrodia camphorata* spore, liver damage, hepatic stellate cells, immunity

## Abstract

Liver fibrosis is a pathological process with intrahepatic diffused deposition of the excess extracellular matrix, which leads to various chronic liver diseases. Drugs with high efficacy and low toxicity for liver fibrosis are still unavailable. *Antrodia camphorata* has antioxidant, antivirus, antitumor and anti-inflammation roles, and has been used to treat liver diseases in the population. However, the hepatoprotective effects of *A. camphorata* spores and the mechanisms behind it have not been investigated. In this study, we evaluate the hepatoprotective effect of spore powder of *A. camphorata* (SP, 100 mg/kg/day or 200 mg/kg/day) on carbon tetrachloride (CCl_4_)-induced liver fibrosis in mice. SP groups reduced serum aspartate aminotransferase (AST) and alanine aminotransferase (ALT) activities compared with the CCl_4_ group. SP also showed a decrease in hydroxyproline (Hyp) content in liver tissues. SP improved cell damage and reduced collagen deposition by H&E, Sirius red and Masson staining. Furthermore, SP down-regulated the mRNA levels of *α-SMA* and *Col 1*, and the protein expression of α-smooth muscle actin (α-SMA), collagen I (Col 1), tumor necrosis factor alpha (TNF-α), toll like receptor 4 (TLR4) and nuclear factor-Κb (NF-κB) p65. In summary, SP has an ameliorative effect on hepatic fibrosis, probably by inhibiting the activation of hepatic stellate cells, reducing the synthesis of extracellular matrix.

## 1. Introduction

Liver fibrosis is the pathological process that results from chronic liver damage caused by multiple reasons. It involves the deposition of excess extracellular matrix (ECM), which compromises the liver functions and is the intermediate step in the development of liver cirrhosis for different chronic liver diseases [1]. Recent studies have indicated that liver fibrosis is a reversible pathological change [2]. It is mainly caused by the activation of hepatic stellate cells (HSCs), which express α-smooth muscle actin (α-SMA) and can be transformed to myofibroblasts and produce massive ECM including collagen I (Col 1) [3]. The prevention and treatment of liver fibrosis mainly include inhibition of HSC activation and proliferation, inhibition of ECM synthesis, promotion of ECM degradation, and induction of activated HSC apoptosis and so on.

Recently, consumers have become more aware of the concept of consuming natural substances containing physiologically active compounds to prevent and treat diseases. Developing effective healthy foods for liver protection is of great significance for preventing and reducing the incidence rate of liver fibrosis, improving the life quality of patients and reducing mortality [4,5,6,7,8,9]. *Antrodia camphorata*, also known as niuzhangzhi, niuzhanggu, and zhangneigu in Chinese, is a unique and rare medicinal mushroom, which was initially found in the forests of Taiwan and has earned the names “Shenzhi” and “forest ruby” [10]. It has a good effect on liver protection, alleviation of a hangover, and detoxification. *A.camphorata* is widely used in southern China. It has been used in Taiwan for more than 500 years. The National Medical Products Administration of China has authorized Fujian Province to manage the production, consumption and import and export of *A. camphorata*. The products of *A. camphorata* have been registered in China as health products (such as No. G20040487). In addition, more than 80 compounds have been isolated from the fruit body and mycelia of *A. camphorata*, including triterpenoids, polysaccharides, maleic acid and succinic acid derivatives, ubiquinone derivatives, adenosine, polypeptides, phenols, sterols, etc. [11,12]. Bioactivity-guided fractionation led to the identification of maleic acid and the succinic acid derivative Antrodin B from the mycelia of *A. camphorata* in submerged culture, which significantly inhibited HSC activation and ECM production [13].

There were a large number of asexual spores produced in the later stage of submerged fermentation of *A. camphorata* under suitable environmental and nutritional conditions [14]. Using its spore as the inoculum in the submerged fermentation of *A. camphorata* has been proved to save lots of time and cost, and improve the controllability and production efficiency of the fermentation process [15]. However, the main components and the function of its spore are largely unknown. In this study, we compared the main components of spore powder, mycelia and cultural broth of *A. camphorata*. Then we evaluated the hepatoprotective effects of spore powder of *A. camphorata* on CCl_4_-induced liver fibrosis in mice and provided scientific evidence for future applications.

## 2. Materials and Methods

### 2.1. Chemicals and Materials

CCl_4_, olive oil, silymarin, hematoxylin, eosin, direct red 80 and Glyceraldehyde-3-phosphate dehydrogenase (GAPDH) Antibody were purchased from Sigma Aldrich (Shanghai, China). SYBR Green Mix and Trizol were obtained from Thermo Fisher Scientific (Waltham, MA, USA). α-SMA Antibody was purchased from Santa Cruz Biotechnology (Santa Cruz, CA, USA). NF-κB P65 Antibody, p-NF-κB p65 Antibody, and F4/80 Antibody were purchased from Cell Signaling Technology (Beverly, MA, USA). All the molecular biology-related reagents not mentioned above were obtained from Takara (Dalian, China). The rest reagents were of analytical grade and were purchased from Sinopharm Chemical Reagent Co., Ltd. (Shanghai, China).

### 2.2. Preparation Spore Powder of A. camphorata

The culture of *A. camphorata* was as previously described [15,16]. In short, *A. camphorata* ATCC 200183 (American Type Culture Collection, Rockville, MD, USA) was maintained on potato dextrose agar (PDA) slants. The mycelia of *A. camphorata* ATCC 200183 were transferred from stock slant to the 500 mL Erlenmeyer flask containing 100 mL culture medium (20 g/L of glucose, 0.5 g/L of yeast extract, 3.0 g/L of KH_2_PO_4_, 0.5 g/L of MgSO_4_, with the initial pH of 5.0), and then incubated for 13 day at 26 °C by shaking at 100 rpm. The fermented product was filtered by six layers of sterile gauze, and the filtrate was *A. camphorata* mycelia and washed three times with sterile water. The supernatant was centrifuged (3000 rpm, 4 °C, 10 min) to obtain the spore, after separating the cultural broth, the precipitate was suspended in deionized water, and then washed three times with sterile water. Spore powder, mycelia and cultural broth of *A. camphorata* were prepared by a Freeze Dry system (Labconco, Kansas City, MO, USA). Spore powder, mycelia and cultural broth of *A. camphorata* were 1.59 g/L, 6.67 g/L and 24.92 g/L, respectively. 

### 2.3. Proximate Analysis and Triterpenoids Analysis

The proximate composition of SP and mycelia of *A. camphorata*, including ash, crude fiber, crude fat and crude protein was determined according to the Chinese National Food Safety standard (GB/T 5009.4-2016, GB/T 5009.10-2003, GB/T 15674-2009, GB/T 5009.5-2016). Total carbohydrate content was determined by the phenol sulfuric acid method. The amount of heavy metals was determined by using atomic absorption spectrophotometer. The hydrolyzed fatty acid was determined according to the standard Determination of Total Fat, Saturated Fat, and Unsaturated Fat in Foods Hydrolytic Extraction–Gas Chromatography (GB/T 22223-2008). Amino acids were identified and quantified by HPLC system (Agilent 1100; Wilmington, DE, USA) with a capillary C18 column (Φ4.0 × 125 mm, Agilent). The mobile phase was 20 mmol/L sodium acetate/methanol/acetonitrile (1:2:2, *v/v/v*). The flow rate was 1.0 mL/min, and the chromatographic peaks were measured at wavelengths of 338 and 262 nm. After extracting triterpenoids of *A. camphorata* spore powder [15], mycelia and cultural broth. Sample solution (20 μL) was applied to HPLC analysis (Chromaster, Hitachi, Japan). The separation was performed in a U-3000 C18 column (Φ4.6 mm × 250 mm, Thermo Fisher Scientific, Waltham, MA, USA), The mobile phase was water-methanol (0 min, 10:90; 10 min, 3:97), the flow rate was 1.40 mL/min and the detection wavelength is 202 nm at 30 °C. Colorimetric method with vanillin–acetic acid system was used to analyze the crude triterpenoids production. The crude polysaccharides, antrodin A and antrodin B were carried out as described previously [15].

### 2.4. Animals and Treatment

A total of 30 male SPF BALB/c mice (6 weeks of age) were obtained from Shanghai SLAC Laboratory Animal Co., Ltd. (Shanghai, China). The mice were divided into 6 groups randomly, with 6 mice per group. Normal control group (CTRL) was injected with olive oil intraperitoneally (every 72 h) and was given 0.5% sodium carboxymethylcellulose by oral gavage (every day) starting from day 1 for 4 weeks. Model group (CCl_4_) was injected with 25% CCl_4_ intraperitoneally (every 72 h) and was given 0.5% sodium carboxymethylcellulose by oral gavage (every day) starting from day 1 for 4 weeks. Positive control drug silymarin group (CCl_4_ + SI) was injected with 25% CCl_4_ intraperitoneally (every 72 h) and was treated with silymarin suspension (100 mg/kg/day) by oral gavage for 4 weeks. Low-dose treatment group (CCl_4_ + SPL) was injected with 25% CCl_4_ intraperitoneally (every 72 h) and was treated with spore powder suspension of *A. camphorata* dissolved in 0.5% sodium carboxymethylcellulose (100 mg/kg/day) by oral gavage for 4 weeks. High-dose treatment group (CCl_4_ + HPL): was injected with 25% CCl_4_ intraperitoneally (every 72 h) and was treated with *A. camphorata* spore powder suspension (200 mg/kg/day) by oral gavage for 4 weeks. The mice were sacrificed 72 h after last time of injection of CCl_4_ or olive oil and were fasted but allowed to drink water for 16 h before sacrifice. The mice were maintained at 22 ± 1 °C with a 12 light and 12 darkness cycle. All mice were fed normal chow diet (Shanghai SLAC Laboratory Animal Co., Ltd., Shanghai, China) ad libitum. The liver tissues were snap-frozen in liquid nitrogen immediately after resection and stored at −80 °C until further analysis. All mouse procedures and protocols were approved by the Institutional Animal Care and Use Committee of Jiangnan University, Wuxi, China [Approval No. JN. No20170319-20170430(42)].

### 2.5. Serum and Tissue Biomakers

Serum aspartate aminotransferase (AST), glutamate aminotransferase (ALT), blood urea nitrogen (BUN), creatinine levels were determined by the corresponding test kits (Shanghai Kehua Bio-engineering Co., Ltd., Shanghai, China). Malondialdehyde (MDA), glutathione (GSH), glutathione peroxidase (GSH-Px) and hydroxyproline (Hyp) levels in liver tissues were determined by commercial kits (Nanjing Jiancheng Bioengineering Research Institute, Nanjing, China) as follows: MDA assay kit (A003-1), GSH assay kit (A006-1), GSH-Px assay kit (A005-1) and Hyp assay kit (A030-2).

### 2.6. Histopathological Study

Fresh liver tissues were immediately immersed in 4% neutral formaldehyde solution at room temperature (RT) for 24 h. The tissues were put into the embedding box and transferred to the dehydrator for dehydration. After soaking the tissue embedding box in the wax tank of the embedding machine for 1 h, the liver tissues were taken out with forceps and put into the embedding bracket with appropriate groove size. The tissues were kept in contact with the bottom surface of the embedding bracket as much as possible. Liquid paraffin was slowly dripped into the box; in order to prevent tissue floating, the embedding box was immediately closed and transferred to the freezing table for freezing. The embedded tissues were fixed in the slot of the slicer and cut into thin sections with a thickness of 4 μM. Liver tissue sections were unfolded in the 42 °C water bath, fixed on pathological slides and baked for 1 h at 70 °C; baked sections were then stained with Sirius red or Masson Stain Kit (D026-1, Nanjing Jiancheng Bioengineering Research Institute, Nanjing, China), sealed and examined under Nikon microscope (Nikon Corporation, Tokyo, Japan). The microscopic images were analyzed by Image J software. Formalin-fixed, paraffin-embedded sections were stained by immunohistochemistry using standard procedures.

### 2.7. RNA Extraction and RT-qPCR Analysis

Total RNA was extracted from the liver tissue with Trizol reagent (Life Technologies). RT-PCR was first performed using PrimeScript^TM^ RT-PCR kit with1 μg of total RNA as the template to synthesize cDNA under the following conditions: 45 °C for 45 min, 95 °C for 5 min, 4 °C for 5 min. qPCR using SYBR Select Master Mix for CFX (Applied Biosystems, Waltham, MA, USA) was followed by using cDNA as the template and *Gapdh* as an endogenous control. Statistical analysis was carried out by using 2^−ΔΔCt^ method. Each RNA sample was tested in triplicates and the average value was taken. Primer sequences for RT–qPCR were: *Gapdh* (forward: 5′- TGG ATT TGG ACG CAT TGG TC-3′, reverse: 5′-TTT GCA CTG GTA CGT GTT GAT-3′), *α-Sma* (*Acta2*) (forward: 5′-GTC CCA GAC ATC AGG GAG TAA-3′, reverse: 5′-TCG GAT ACT TCA GCG TCA GGA-3′), *Col 1* (forward: 5′-GGT GAG CCT GGT CAA ACG G-3′, reverse: 5′-ACT GTG TCC TTT CAC GCC TTT-3′).

### 2.8. Protein Extraction and Western Blot Analysis

The liver tissue was homogenized in RIPA buffer (Cell Signaling Technology, Danvers, MA, USA). The homogenates were centrifuged at 14,000× *g* for 15 min at 4 °C, and the supernatant was collected. The protein concentrations were calculated using a BCA Protein Assay Kit (Thermo Fisher Scientific, Waltham, MA, USA). The samples of equal proteins were separated on 10% polyacrylamide gel, transferred to membrane, sealed, incubated with corresponding first antibodies (Col 1, α-SMA, TLR4, TNF-α and GAPDH) and second antibodies (HRP-second antibodies). The blots were visualized using the ECL chemiluminescence kit (Thermo Fisher Scientific, Waltham, MA, USA). Image J software (National Institutes of Health, Bethesda, MD, USA) was used for densitometric analysis of the bands.

### 2.9. Statistical Analysis

Results are presented as mean ± SEM. All the data were analyzed by one-way analysis of variance (ANOVA) and Tukey’s test for multiple comparisons. All *p* value of less than 0.05 was considered statistically significant. Data analysis was processed by GraphPad Prism 7 software (La Jolla, CA, USA).

## 3. Results

### 3.1. Chemical Characteristics of Spore, Mycelia and Cultural Broth of A. camphorata

**Figure 1:**
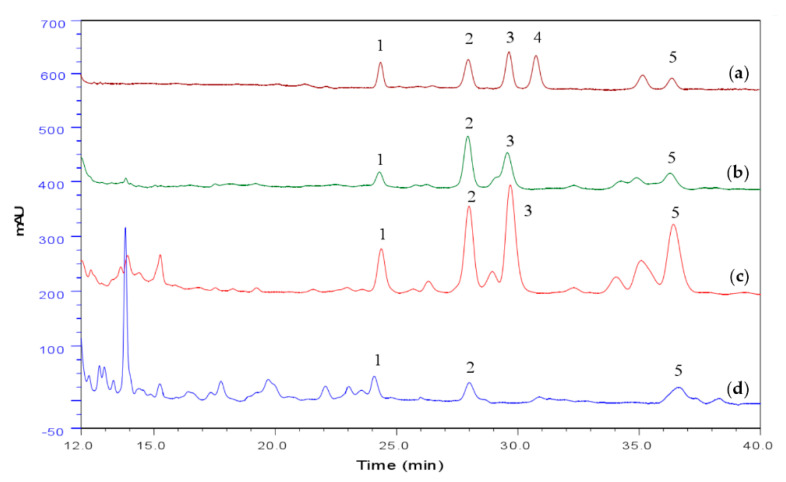
HPLC chromatogram of triterpenoids of *A. camphorata* in submerged culture, including the standard (**a**), mycelia (**b**), spore (**c**), and culture broth (**d**). 1: Ergosterol; 2: Cholesterol; 3: Lanosterol; 4: Stigmasterol; 5: Sitosterol.

Proximate compositions in spore, mycelia and cultural broth of *A. camphorata* are presented in Table S1. There were significant differences in both ash and fat content among the spore, mycelia and cultural broth of *A. camphorata*, but the protein and carbohydrate contents did not differ statistically. The 1.03% ash content in the spore was two times higher than in the cultural broth of the *A. camphorata*. The crude polysaccharides in spore of *A. camphorata* is 13.10%, which is lower than 16.23% in mycelia. The fermentation product of *A. camphorata* contains eight essential mineral elements, including potassium (K), calcium (Ca), sodium (Na), magnesium (Mg), iron (Fe), manganese (Mn), zinc (Zn), and copper, which are indispensable to the human body (Table S2). The content of Na in spore of *A. camphorata* was about 8-fold that in mycelia, and the content of K and Mg was 4-fold that in mycelia. The content of Ca and Cu were 1.82-fold and 1.48-fold of mycelia. Relative contents of saturated fatty acids in spore, mycelia and culture broth were 58.62%, 55.32% and 60.79%, respectively. In addition, for unsaturated fatty acids, these values were 43.15%, 40.52% and 39.18%, respectively (Table S3). In terms of amino acid composition (Table S4), the content of total and essential amino acids in spore of *A. camphorata* was significantly higher than that in mycelia and cultural broth. Among them, the content of glutamic acid (Glu) and tyrosine (Trp) in spore was 1.41 and 1.46-fold those in mycelia. The content of serine (Ser), glycine (Gly) and proline (Pro) in spore is more than 1.2-fold that in mycelia. Triterpenoids are the major active compounds of *A. camphorata* and have been reported to possess potent hepatoprotective activities. We used the vanillin-perchloric acid method to determine total triterpenoids and HPLC analysis to determine the main triterpenoids of *A. camphorata*. The results showed that the content of total triterpenoids and the main triterpenoids of *A. camphorata* spores were higher than that of mycelia and culture broth (Table 1 and Figure 1). In addition, the content of antrodin A and antrodin B of *A. camphorata* spore were 2.49-fold and 1.53-fold of mycelia (Figure S1 and Table S5).

### 3.2. Effects of Spore Powder of A. camphorata on Serum and Tissue Biomarkers in CCl_4_-Induced Liver Fibrosis in Mice

**Figure 2:**
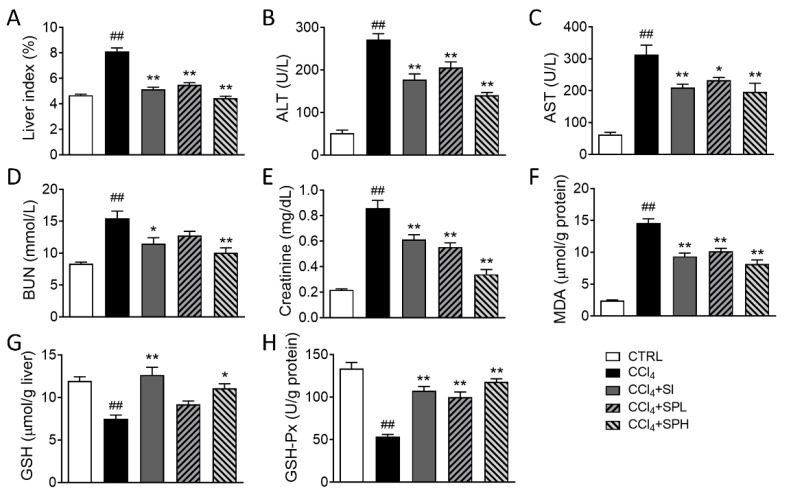
Effect of spore powder of *A. camphorata* on the serum and tissue biomarkers in CCl_4_-treated mice. (**A**) Liver index. (**B**) Serum ALT level. (**C**) Serum AST level. (**D**) Serum BUN level. (**E**) Serum creatinine level. (**F**) MDA level of liver tissues. (**G**) GSH level of liver tissues. (**H**) GSH-Px level of liver tissues. Data are presented as mean ± SEM (*n* = 6). Different letters indicate significantly different values according to a one-way ANOVA using Tukey’s test for multiple comparisons. Compared with control group (CTRL), ^##^ *p* < 0.01; compared with CCl_4_ induced group (CCl_4_), * *p* < 0.05, ** *p* < 0.01. CCl_4_, carbon tetrachloride; ALT, alanine aminotransferase; AST, aspartate aminotransferase; BUN, blood urea nitrogen; MDA, malondialdehyde; GSH, glutathione; GSH-Px, glutathione peroxidase; SI, silymarin; SPL, low-dose treatment with spore powder of *A. camphorata*; SPH, low-dose treatment with spore powder of *A. camphorata*.

ALT and AST are the most commonly used biomarkers to evaluate the liver functions. When liver cells are damaged, the activity of ALT and AST in serum increases, especially in liver and gall diseases. As shown in the Figure S2 and Table S6, in the preliminary experiment, the subacute toxicity of SP (50, 500, 2000 and 5000 mg/kg) was examined on BALB/c mice by oral gavage daily for 28 days. Compared with the control group, there were no significant changes on body weight, serum ALT, AST, and organ coefficient. Then, to evaluate the hepatoprotective effects of spore powder of *A. camphorata*, we used CCl_4_ to induce liver fibrosis in mice. As shown in Figure 2, the activity of ALT and AST levels in the CCl_4_ group after CCl_4_ induction were significantly higher compared with the CTRL group (^##^ *p* < 0.01). After treatments with different doses of spore powder of *A. camphorata* (SPL 100 mg/kg and SPH 200 mg/kg) and positive control drug silymarin (SI 100 mg/kg). The activity of ALT and AST in mice decreased significantly (** *p* < 0.01). In addition, the effect of lowering ALT was better in CCl_4_ + SPH group compared with positive control and CCi_4_ + SPL group. Besides, spore powder of *A. camphorata* (SPH 200 mg/kg) protected kidney function and displayed antioxidant activity, such as significantly cause decrease in serum BUN and serum creatinine, significantly down-regulate MDA level of liver tissues, and up-regulate the GSH and GSH-Px levels of liver tissues in CCl_4_-induced mice. These results show that *A. camphorata* spores have an anti-hepatic injury effect.

### 3.3. Effects of Spore Powder of A. camphorata on Liver Pathology on CCl_4_-Induced Liver Fibrosis in Mice

**Figure 3:**
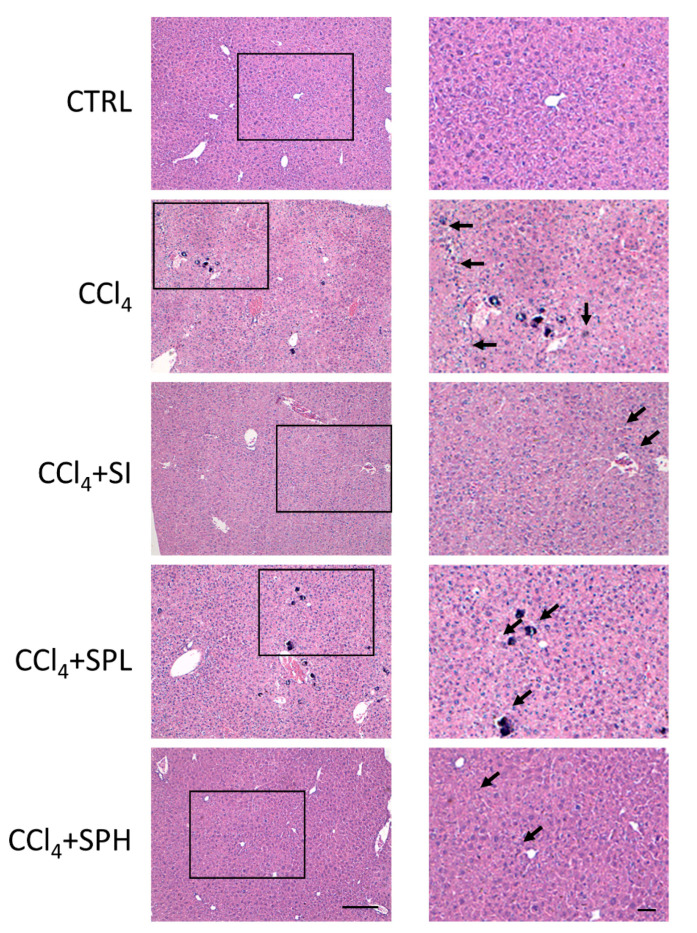
Effect of spore powder of *A. camphorata* on liver pathology in CCl_4_ treated mice by H&E staining. Scale bar: 100 μm. CTRL, control group; CCl_4_, carbon tetrachloride; SI, silymarin; SPL, low-dose treatment with spore powder of *A. camphorata*; SPH, low-dose treatment with spore powder of *A. camphorata*.

The liver pathological examination was done using H&E staining. The liver tissue of the CTRL group was closely arranged, the structure of the liver lobule was clear, and the size of the liver cells was normal without degeneration or necrosis (Figure 3). CCl_4_ induced hepatocyte necrosis that was presented by nuclear shrinkage, nuclear membrane disappearance, nucleolar fragmentation and inflammatory cell infiltration (Figure 3). In the CCl_4_ + SPL group, the condition of hepatocyte necrosis was slightly improved, while in the high dose group, the hepatoprotective effect was significant, and was similar to that of CCl_4_ + SI group.

### 3.4. Effects of Spore Powder of A. camphorata on Collagen Content in LiverTissues 

**Figure 4:**
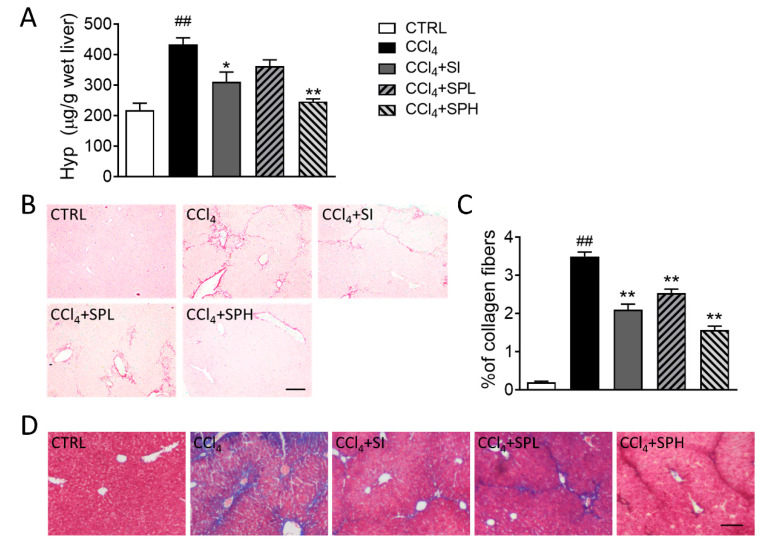
Effects of spore powder of *A. camphorata* on collagen accumulation in the liver tissues in CCl_4_-treated mice. (**A**) Hydroxyproline content (Hyp) in liver tissues from mice of different groups. (**B**) Sirius red staining and collagen content (**C**) in liver tissues from mice of different groups. Masson Trichrome staining (**D**) in liver tissues from mice of different groups. Data are presented as mean ± SEM (*n* = 6). Different letters indicate significantly different values according to a one-way ANOVA using Tukey’s test for multiple comparisons. Compared with control group (CTRL), ^##^ *p* < 0.01; compared with CCl_4_-induced group (CCl_4_), * *p* < 0.05, ** *p* < 0.01. Scale bar: 100 μm. Hyp, hydroxyproline; CCl_4_, carbon tetrachloride; SI, silymarin; SPL, low-dose treatment with spore powder of *A. camphorata*; SPH, low-dose treatment with spore powder of *A. camphorata*.

During the pathogenesis of liver fibrosis, collagen is accumulating in hepatocytes. Hydroxyproline (HYP) is a characteristic amino acid in collagen [17]. Its content can indirectly reflect the level of collagen accumulation in the liver and therefore is an indicator to evaluate the degree of liver fibrosis. After CCl_4_ induction, Hyp level in the liver tissues significantly increased (*p* < 0.01), which indicated that the liver fibrosis model was established successfully (Figure 4A). Compared with CCl_4_ group, Hyp level in CCl_4_ + SPH group significantly decreased (*p* < 0.01). This result indicated that 200 mg/kg *A. camphorata* spore powder could significantly lower the Hyp level in the livers of mice with liver fibrosis and inhibit the deposition of collagen. Sirius red staining and Masson Trichrome staining were also used to stain collagen of specific liver tissues (Figure 4B–D). Collagen deposition appeared in the liver sections, which spread out around the blood vessels in CCl_4_ induced liver fibrosis in mice. After silymarin treatment, the collagen level in the liver tissues significantly decreased (*p* < 0.05), indicating that the degree of liver fibrosis also decreased (Figure 4B,C). Compared with the CCl_4_ group, liver fibrosis was improved, and the collagen level significantly decreased (*p* < 0.01) in two treatment groups, especially in the CCl_4_ + SPH group, which was close to that of CTRL group. The results were consistent with the results of Hyp in the liver tissues. 

### 3.5. Effects of Spore Powder of A. camphorata on α-SMA and Col 1 Expression in Liver Tissues

**Figure 5:**
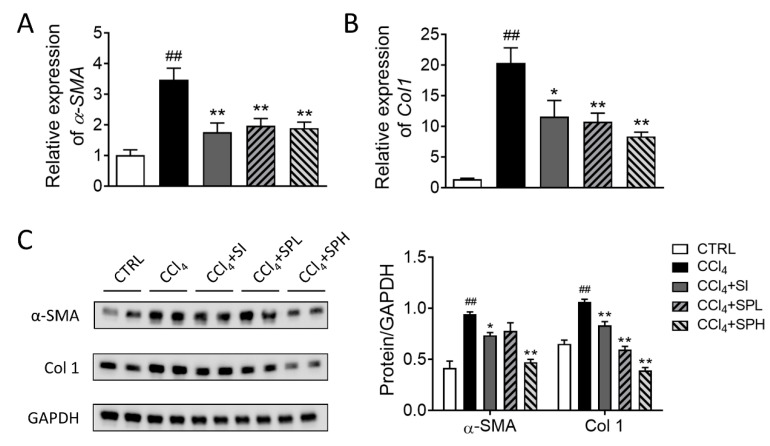
Effects of spore powder of *A. camphorata* on the expression of α-SMA and Col1 in liver tissues from mice treated by CCl_4_. The transcription levels of *α-SMA* (**A**) and *Col 1* (**B**) genes in the liver tissues. (**C**) The translation levels of *α-SMA* and *Col 1* genes in the liver tissues. Data are presented as mean ± SEM (*n* = 6). Different letters indicate significantly different values according to a one-way ANOVA using Tukey’s test for multiple comparisons. Compared with control group (CTRL), ^##^ *p* < 0.01; compared with CCl_4_-induced group (CCl_4_), * *p* < 0.05, ** *p* < 0.01. α-SMA, α-smooth muscle actin; Col 1, collagen I; GAPDH, Glyceraldehyde-3-phosphate dehydrogenase. CTRL, control group; CCl_4_, carbon tetrachloride; SI, silymarin; SPL, low-dose treatment with spore powder of *A. camphorata*; SPH, low-dose treatment with spore powder of *A. camphorata*.

The expression of genes related to liver fibrosis was measured at the molecular level. *α-SMA* is the marker gene of HSCs activation and Col 1 is the main component of ECM. After CCl_4_ induction, the transcription and translation levels of *α-SMA* and *Col 1* genes in the liver tissues were significantly elevated (^##^ *p* < 0.01), and ECM was accumulated in large quantities. Compared with the CCl_4_ group, the transcription and translation of *α-SMA* and *Col 1* genes were significantly inhibited in two SP treatment groups. In addition, the effect of SP on translational level was better than that of silymarin (Figure 5).

### 3.6. Effects of Spore Powder of A. camphorata on TNF-α, TLR4, NF-κB and F4/80 Protein Expression in Liver Tissues

**Figure 6:**
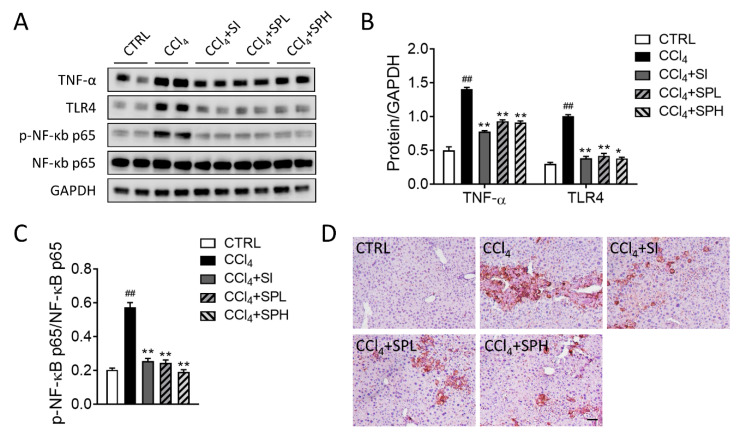
Effects of spore powder of *A. camphorata* on the expression of TNF-α, TLR4, NF-Κb (**A**–**C**) and the immunohistochemistry staining of F4/80 (**D**) in liver tissues from mice treated by CCl_4_. Data are presented as mean ± SEM (*n* = 6). Different letters indicate significantly different values according to a one-way ANOVA using Tukey’s test for multiple comparisons. Compared with control group (CTRL), ^##^ *p* < 0.01; compared with CCl_4_-induced group (CCl_4_), * *p* < 0.05, ** *p* < 0.01. TNF-α, tumor necrosis factor alpha; TLR4, toll like receptor 4; NF-Κb, nuclear factor-Κb; CTRL, control group; CCl_4_, carbon tetrachloride; SI, silymarin; SPL, low-dose treatment with spore powder of *A. camphorata*; SPH, low-dose treatment with spore powder of *A. camphorata*.

The protein expression of tumor necrosis factor alpha (TNF-α) and Toll like receptor 4 (TLR4) significantly increased (^##^ *p* < 0.01) in CCl_4_-induced liver fibrosis in mice by Western blotting (Figure 6A,B). TNF-α expression significantly decreased in two treatment groups (** *p* < 0.01). TLR4 expression also significantly decreased in two treatment groups (** *p* < 0.01). In addition, the expression level in CCl_4_ + SPH group was lower compared with CCl_4_ + SPL and CCl_4_ + SI group. NF-kB is the major regulator in the immune pathway. As shown in Figure 6A,C, we detected a significant increase in the phosphorylation of NF-κB p65 levels in CCl_4_ group compared with the CTRL group, suggesting the activation of NF-κB signaling pathway. After treatment with SP, there was a marked reduction of NF-κB protein phosphorylation levels in the liver tissue. Additionally, liver histology showed that F4/80^+^ cells were also increased in the liver tissue in CCl_4_-induced mice, and were reduced by SP treatment (Figure 6D).

## 4. Discussion

In this study, we analyzed and compared the components of spore and mycelium powder of *A. camphorata*. The content of total triterpenoids and the main triterpenoids of spore were higher than that of mycelia. Then, we evaluated the hepatoprotective effects of spore powder of *A. camphorata* on CCl_4_-induced liver fibrosis in mice and found that the degree of liver injury and liver fibrosis in mice treated with *A. camphorata* spore was significantly improved compared with the model group (CCl_4_), which indicated that *A. camphorata* spore could improve CCl_4_ induced liver injury in mice.

*A. camphorata* has been studied for many functions, including detoxification, fat-removal, hepatoprotection, heart strengthening, kidney tonifying, immune regulation, gut regulation, pain releasing, anti-bacteria, anti-inflammation and anti-cancer [18]. Recent studies have found that *A. camphorata* is rich in a variety of active compounds such as polysaccharides, triterpenoids, adenosine, polypeptides, vitamins, trace elements, nucleic acids, and sterols [11]. The ergostane-type triterpenoids was characteristic constituents of *A. camphorata* [19]. The ergostane-type triterpenoids (Antcin B, Antcin K and Antcin H) could protect against liver injury in vivo or vitro. Among them, antcins B and K ameliorated CCl_4_-induced liver injury in mice, decreasing ALT and AST levels and the incidence of liver necrosis [20,21]. Cai et al. [22] showed that the polysaccharides from *A. camphorata* in submerged culture could alleviate the hydrogen peroxide induced DNA damage in Chang liver cells. The ethyl acetate extract of *A. camphorata* was shown to induce the activation of calpain-3 (CAPN-3) and caspases-12 (CASP-12) by increasing the level of Ca^2+^ in the cytoplasm of Hep3B cells and further induced the apoptosis of hepatoma cells [23]. The crude extract of the fermented products and fermented filtrate from *A. camphorata* in submerged culture has been proved to improve the liver injury and fibrosis induced by CCl_4_, as well as the liver fibrosis induced by dimethylnitrosamine [24,25]. He et al. [26] evaluated the hepatoprotective effect of ethanol extract from large-scale submerged fermentation of *A. camphorata* in vitro and found that the extract could effectively reduce the ethanol induced damage in liver AML12 cells. Lin et al. [27] found that *A. camphorata* inhibits the elevated liver malondialdehyde level caused by alcohol, and improve the activities of glutathione, glutathione reductase and glutathione peroxidase to prevent alcoholic liver disease. 

ECM is a very important part of the liver structure. Its synthesis and degradation are maintained in dynamic equilibrium, in general. When suffering from chronic persistent damage, the synthesis of liver ECM increases. Excessive ECM is very likely to cause liver fibrosis, which eventually leads to liver failure and is life-threatening. Therefore, liver fibrosis is the imbalance of ECM synthesis and degradation [28]. Our study showed that the transcription and translation levels of *α-SMA* and *Col 1* in the liver tissues of mice were significantly inhibited after treatments of SP, and the translation level was dose-dependent. The results of pathological examination also suggested that SP improves the liver fibrosis induced by CCl_4_ in mice.

Immune response plays an important role in the formation of liver fibrosis. Activated HSCs secrete inflammatory cytokines, which cannot only interact directly with immune cells by expressing different adhesion molecules but also regulate the adaptive immune system by acting as antigen-presenting cells [29]. Cells that regulate the progression of liver fibrosis include liver sinusoidal endothelial cells (LSEC), Kupffer cells, T cells and other types of inflammatory cells [17]. Activation of Kupffer cells leads to increased NF-κB activity and secrets proinflammatory cytokines or chemokines similar to TNF-α [30]. TNF-α can induce neutrophil infiltration and stimulate mitochondria of apoptotic hepatocytes to generate oxidants, which further aggravate liver fibrosis [31]. TLRs are involved in innate immunity and help connect innate and adaptive immunity. HSCs are regulated by TLR4 on their own surface. TLR4 can activate NF-κB and JNK, promote the expression of IL-8 and MCP-1, enhance the inflammatory response and promote liver fibrosis [32,33]. The water extract of *A. camphorata* ameliorates high-fat diet-induced obesity in mice by maintaining intestinal integrity, regulating intestinal microbiota to reduce fat accumulation and serum TG level and reversing inflammatory factors such as IL-1β, IL-6 and TNF-α [34]. Our study indicated that SP effectively reduces the expression of TNF-α protein in liver fibrosis in mice induced by CCl_4_.

## 5. Conclusions

In conclusion, spore powder of *A. camphorata* contains high content of total triterpenes, and the main triterpenoids and could improve CCl_4_-induced liver fibrosis in BLAB/c mice, probably through inhibiting the activation of hepatic stellate cells, reducing the synthesis of extracellular matrix and regulating the immune responses.

## Figures and Tables

**Table 1:** Triterpenoids content of spore, mycelia and cultural broth of *A. camphorata*.

	Spore (g/100 g)	Mycelia (g/100 g)	Cultural Broth (g/100 g)
HPLC	1.31	1.07	0.00051
vanillin–acetic acid system	1.68	1.59	0.097

## Supplementary Materials

The following are available online at https://www.mdpi.com/2072-6643/12/9/2778/s1, Figure S1: Chromatograms of antrodin standards and methanol extract of *A. camphorata* spore. (A) standard of antrodin A; (B) standard of antrodin B; (C) methanol extract of *A. camphorata* spore; (D) methanol extract of *A. camphorata* mycelia, Figure S2: Body weight change in mice during spore powder of *A. camphorata* by oral gavage daily for 28 days. Data are expressed as means ± SD (n = 5), Table S1: Proximate composition of spore, mycelia and cultural broth of *A. camphorata*, Table S2: Mineral contents of spore, mycelia and cultural broth of *A. camphorata*, Table S3: Fatty acid composition of spore and mycelia of *A. camphorata*, Table S4: Amino acid composition of spore and mycelia of *A. camphorata*, Table S5: Antrodin A and antrodin B content of spore and mycelia of *A. camphorata*, Table S6: The organ coefficient results of mice treated orally with spore powder of *A. camphorata* daily for 28 days. Data are expressed as means ± SEM (n = 5).
