# Supplementary material for: Protective Effect of Spore Powder of Antrodia camphorata ATCC 200183 on CCl4-Induced Liver Fibrosis in Mice
Source: Nutrients. 2020 Sep 11;12(9):2778. doi: 10.3390/nu12092778 (PMC7551437; doi:10.3390/nu12092778)
Supplement: Supplementary file 1 [file nutrients-12-02778-s001.zip › nutrients-921864-supplementary.pdf]

Supplementary Table 1. Proximate composition of spore, mycelia and cultural broth of *A. camphorata*

Component (g/100g)	Spore	Mycelia	Cultural broth
Ash	1.03	1.79	0.59
Fiber	7.74	8.26	nd[1]
Fat	2.79	2.07	0.13
Protein	36.88	33.32	33.31
Carbohydrate	51.89	54.56	65.81
Crude polysaccharide	13.10	16.23	nd[1]

[1] Not detected.

Supplementary Table 2. Mineral contents of spore, mycelia and cultural broth of *A. camphorata*

Content (mg/100g)	Spore	Mycelia	Cultural broth
K	3460	747	863.56
Na	447	55.8	59.39
Ca	112	61.4	93.50
Mg	1210	148	284.11
Cu	0.34	0.23	nd[1]
Mn	0.74	0.77	0.47
Fe	11.3	13	17.66
Zn	3.37	3.98	0.30
Pb	0.021	0.022	nd[1]
As	5.4×10^{-3}	2.4×10^{-3}	4.8×10^{-3}

[1] Not detected.

Supplementary Table 3. Fatty acid composition of spore, mycelia and cultural broth of *A. camphorata*

Content	Relative content of fatty acid (%)		
	Spore	Mycelia	Cultural broth
Palmitic acid ($C_{16}H_{32}O_2$)	21.52	22.28	41.04
Palmitoleic acid ($C_{16}H_{30}O_2$)	1.49	1.17	0.98
Stearic acid ($C_{18}H_{36}O_2$)	37.10	33.04	20.78
Oleic acid ($C_{18}H_{34}O_2$)	21.92	20.35	22.67
Linoleic acid ($C_{18}H_{32}O_2$)	14.32	18.09	14.50
Linolenic acid ($C_{18}H_{30}O_2$)	2.79	3.54	1.03

Supplementary Table 4. Amino acid composition of spore, mycelia and cultural broth of *A. camphorata*

Content (g/100g)	Spore	Mycelia	Cultural broth
Asp	2.09	2.3	5.23×10^{-2}
Glu	4.41	3.12	1.01×10^{-1}
Ser	1.75	1.41	2.75×10^{-2}
His	0.69	0.63	8.23×10^{-3}
Gly	1.76	1.4	1.59×10^{-1}
Thr[1]	1.51	1.27	1.60×10^{-2}
Arg	2.32	2.15	5.30×10^{-2}
Ala	3.24	3.04	6.83×10^{-2}
Tyr	0.88	0.77	8.00×10^{-3}
Cys	0.17	0.24	9.87×10^{-4}
Val[1]	1.58	1.39	2.28×10^{-2}
**Met[1]**	**0.51**	**0.46**	**5.87×10^{-3}**
Phe[1]	1.57	1.44	1.78×10^{-2}
Ile[1]	1.14	0.98	1.34×10^{-2}
Leu[1]	2.42	2.13	3.07×10^{-2}
Lys[1]	1.82	1.59	2.89×10^{-2}
Pro[1]	2.32	1.91	1.00×10^{-1}
Trp[1]	0.38	0.26	Nd[2]
Total amino acids	30.56	26.49	7.14×10^{-1}
Essential amino acids	10.93	9.52	1.35×10^{-1}

[1] Essential amino acid. [2] Not detected.

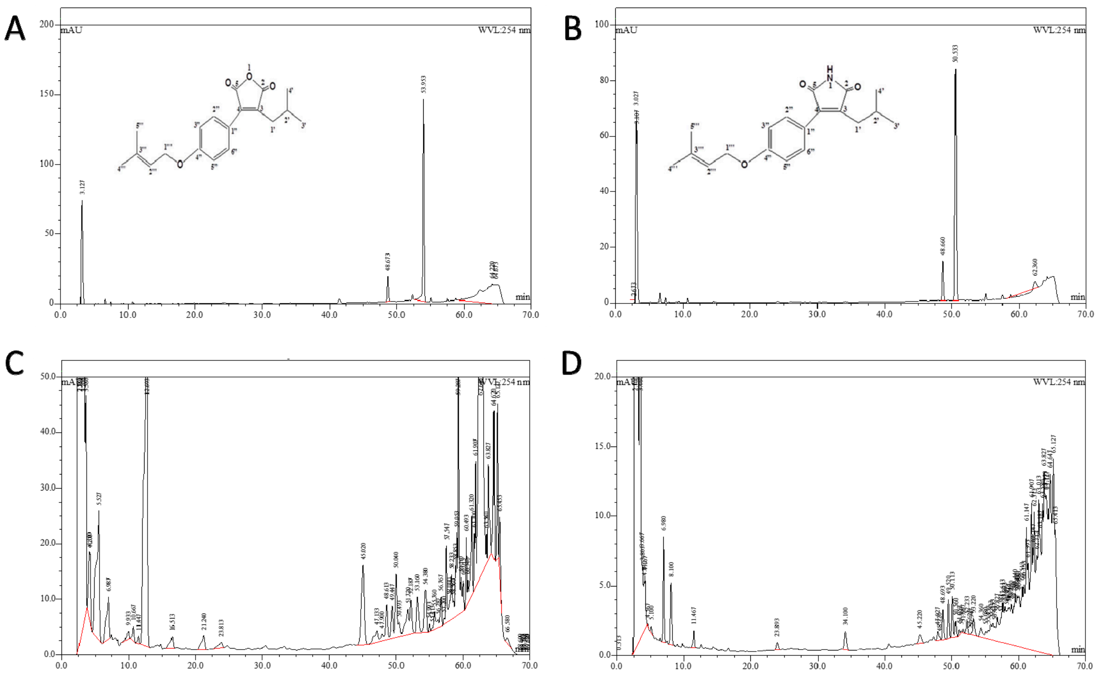

Supplementary Figure 1. Chromatograms of antrodin standards and methanol extract of *A. camphorata* spore. (A) standard of antrodin A; (B) standard of antrodin B; (C) methanol extract of *A. camphorata* spore; (C) methanol extract of *A. camphorata* mycelia.

Supplementary Table 5. Antrodin A and antrodin B content of spore and mycelia of *A. camphorata*

Content (mg/kg)	Spore	Mycelia
antrodin A	0.7865	0.3158
antrodin B	1.8722	1.2221

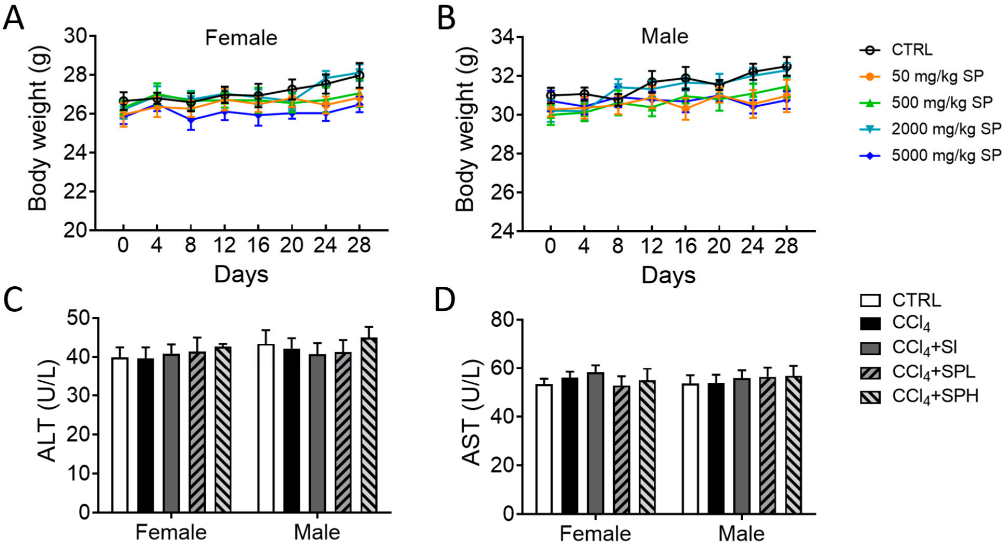

Supplementary Figure 2. Body weight change in mice during spore powder of *A. camphorata* by oral gavage daily for 28 days. Data are expressed as means ± SD (n = 5).

Supplementary Table 6. The organ coefficient results of mice treated orally with spore powder of *A.*

camphorata daily for 28 days. Data are expressed as means ± SEM (n = 5).

Parameters	Control	50 mg/kg SP	500 mg/kg SP	2000 mg/kg SP	5000 mg/kg SP
Female					
Heart (g/100 g)	0.53±0.06	0.57±0.05	0.49±0.04	0.55±0.04	0.52±0.04
Liver (g/100 g)	5.42±0.17	4.29±0.07	5.39±0.48	5.51±0.24	5.40±0.16
Spleen (g/100 g)	0.32±0.03	0.20±0.05	0.39±0.04	0.32±0.01	0.31±0.04
Lung (g/100 g)	0.52±0.07	0.63±0.03	0.59±0.03	0.51±0.05	0.60±0.03
Kidney (g/100 g)	1.25±0.05	1.19±0.05	1.14±0.08	1.21±0.05	1.12±0.06
Brain (g/100 g)	1.32±0.11	1.17±0.21	0.97±0.09	1.56±0.06	1.16±0.08
Stomach (g/100 g)	0.89±0.08	0.95±0.06	0.87±0.02	0.96±0.10	0.86±0.09
Thymus (g/100 g)	0.39±0.02	0.37±0.06	0.31±0.02	0.27±0.05	0.31±0.01
Male					
Heart (g/100 g)	0.63±0.06	0.64±0.06	0.63±0.01	0.60±0.03	0.46±0.04
Liver (g/100 g)	5.32±0.23	5.03±0.21	5.30±0.21	0.52±0.19	4.48±0.23
Spleen (g/100 g)	0.28±0.03	0.28±0.03	0.33±0.01	0.31±0.01	0.26±0.02
Lung (g/100 g)	0.59±0.08	0.62±0.04	0.68±0.05	0.61±0.03	0.53±0.01
Kidney (g/100 g)	1.37±0.19	1.34±0.18	1.70±0.03	2.02±0.09	1.51±0.08
Brain (g/100 g)	0.97±0.13	1.19±0.06	1.10±0.03	1.08±0.04	1.08±0.06
Stomach (g/100 g)	0.98±0.28	0.82±0.12	0.96±0.09	1.00±0.02	0.78±0.07
Thymus (g/100 g)	0.28±0.03	0.23±0.07	0.23±0.03	0.24±0.01	0.28±0.03
